# Persistent *Staphylococcus aureus* Colonization Is Not a Strongly Heritable Trait in Amish Families

**DOI:** 10.1371/journal.pone.0017368

**Published:** 2011-02-28

**Authors:** Mary-Claire Roghmann, J. Kristie Johnson, O. Colin Stine, Alison D. Lydecker, Kathleen A. Ryan, Braxton D. Mitchell, Alan R. Shuldiner

**Affiliations:** 1 University of Maryland School of Medicine, Baltimore, Maryland, United States of America; 2 VA Maryland Health Care System, Baltimore, Maryland, United States of America; University of Iowa, United States of America

## Abstract

About 20% of adults are persistently colonized with *S. aureus* in the anterior nares. Host genetic factors could contribute susceptibility to this phenotype. The objective of this study was to determine whether the phenotype of persistent *S. aureus* colonization aggregates in family members who live in different households. Healthy adults and their eligible same sex siblings who lived in different households were recruited from the Old Order Amish of Lancaster, Pennsylvania. All participants had two cultures of the anterior nares to determine if they were persistently colonized with *S. aureus*. Three hundred and ninety eight participants finished the study, of whom 166 were index cases and 232 were siblings of index cases. Eighteen per cent (71/398) of all participants and 17% (29/166) of index cases were persistently colonized with *S. aureus*. Twenty two per cent (8/36) of siblings of persistently colonized index cases were persistently colonized with *S. aureus* compared to 17% (34/196) of siblings of non-persistently colonized index cases, yielding a prevalence rate ratio of 1.28 (95% CI: 0.65–2.54, p = 0.64) and sibling relative risk of 1.25 (95% CI: 0.65–2.38, p = 0.51). The heritability of persistent colonization was 0.19±0.21 (p = 0.31). Persistent *S. aureus* colonization does not strongly aggregate in Amish family members in different households and heritability is low, suggesting that environmental factors or acquired host factors are more important than host genetic factors in determining persistent *S. aureus* colonization in this community.

## Introduction

*Staphylococcus aureus* is an opportunistic bacterial pathogen and a common cause of both community and hospital acquired infections. These infections have become progressively more difficult to treat due to development of resistance to beta-lactam antibiotics and the number of infections due to methicillin-resistant *S. aureus* (MRSA) has increased [1]–[8]. A major obstacle to developing new preventive measures is that relatively little is known about the host determinants of *S. aureus* carriage or colonization. Understanding the determinants of *S. aureus* colonization could lead to new approaches to prevent *S. aureus* infections, including those caused by MRSA.

Colonization plays a key role in development of *S. aureus* infections [9]. Nasal colonization or carriage with *S. aureus* is common and most often precedes *S. aureus* infection; in fact, rates of infection are three-fold higher in nasal carriers. Infected individuals are most commonly infected with strains found in their anterior nares [10], which are the primary ecological niche for *S. aureus*. Importantly, elimination of colonization at the anterior nares can lead to a temporary decrease in infections in certain populations [11]. In addition, people with *S. aureus* colonization can serve as a source of transmission to others.

Longitudinal studies of *S. aureus* colonization of the anterior nares have demonstrated three carrier patterns in healthy adult populations. About 20% (range 12–30%) of individuals are persistent carriers, about 30% are intermittent carriers (range 16–70%) and about 50% are persistent non-carriers (range 16-69%) [9]. The most widely used definition of persistent carriage is a positive cultures from ≥80% of 10 weekly cultures. Persistent carriers typically carry a single bacterial strain at high levels of colonization over time, shed high levels of *S. aureus* into the environment and are at higher risk of infection than intermittent or non carriers. In contrast, intermittent carriers carry different strains, one strain at a time, at lower levels of colonization over time.

Host genetic characteristics may contribute to nasal carriage. Biological evidence supports the hypothesis that host factors could influence persistent colonization by determining the immune response to *S. aureus* or adherence of *S. aureus* to the nasal epithelium [12], [13]. Persistent *S. aureus* carriers are more likely to reacquire colonization with their original *S. aureus* strain after decolonization and subsequent artificial inoculation of a mixture of *S. aureus* strains including their own, while non-carriers subjected to artificial inoculation return to non-colonization [14]. A familial predisposition to nasal carriage was reported from a large community-based prevalence study in the 1960’s, [15]; however, two twin studies were inconclusive [16], [17]. There have been a number of genetic association studies for persistent *S. aureus* colonization using a candidate gene approach. Investigators from the Netherlands phenotyped almost 4000 adults for persistent *S. aureus* colonization status and tested for associations with polymorphisms in specific genes associated with the host inflammatory response. Their findings were mixed with some modestly positive and other negative associations [18]–[22]. None of the significant associations have been replicated, perhaps, due to the paucity of studies on the subject. Thus, although the evidence is suggestive, there remains insufficient evidence either to confirm or refute a host genetic contribution to persistent *S. aureus* colonization.

The objective of this study was to determine whether the phenotype or trait of persistent *S. aureus* colonization aggregates in family members in different households. Specifically, we compared the prevalence of persistent *S. aureus* colonization of the anterior nares between siblings of adults who were colonized with persistent S. *aureus* colonization and siblings of adults who were not colonized. The study was carried out in Amish adults from the Old Order Amish community in Lancaster County, Pennsylvania.

## Methods

### Study Design

This was a prospective, observational study of healthy, Old Order Amish adults and their same-sex eligible siblings to determine if the trait of persistent *S. aureus* colonization aggregates among family members living in different households. The Old Order Amish have large, geographically localized families, making them an ideal population for studies of familial aggregation, especially those such as this one involving adult siblings living in different households.

Our study population consisted of a convenience sample of healthy, Old Order Amish adults (n = 166 index cases) and as many same sex adult siblings of each index case whom we could subsequently recruit (n = 232 siblings). We used a simple cohort design to assess familial aggregation, comparing the prevalence of persistent *S. aureus* colonization in an exposed group (siblings of index cases who were persistent colonizers) with prevalence in an unexposed group (siblings of index cases who were not persistent colonizers). We reasoned that a higher prevalence of persistent colonization observed in siblings of colonizers would provide evidence for familial aggregation.

All recruitment was performed between March 2008 and October 2009. All study participants lived in or near Lancaster County, PA. Individuals were not eligible for the study if they were less than 18 years of age, had active skin lesions, diabetes, end-stage renal disease, or had taken antibiotics in the last 30 days because these factors are associated with increased *S. aureus* colonization. The 166 index cases were primarily recruited by re-contacting Amish participants who had participated as healthy volunteers in one or more other studies in the past at the Amish Research Clinic. Once an index case was identified, we then recruited as many same-sex eligible siblings as we could, provided that they lived in a different household as defined by a different street address. The requirement that siblings reside in different households was made to minimize sharing of environment as a source for concordance of colonization rates.

Basic demographic (date of birth, gender, household population, contact with animals) and health history (history of skin conditions and recent surgeries, hospitalizations and antibiotic use) information was collected at the enrollment visit. All study data was entered into a relational database. Quality control was performed on a quarterly basis via logic checks on the entirety of the database and comparison of source documentation to the database values for 10% of the participants.

The protocol was approved by the University of Maryland Baltimore IRB. An Amish community liaison accompanied research staff during each study visit to ensure cultural sensitivity. Informed consent was obtained in writing from all participants.

### Microbiology Methods

A trained research nurse first obtained two cultures of the anterior nares from each participant. Swabs were aseptically inserted into the front of the nostril and rotated twice. Using the same swab, the process was repeated in the other nostril. These first two cultures were obtained between one and six weeks apart, were shipped overnight from Lancaster County to the University of Maryland, Baltimore, and were semi-quantitatively tested for *S. aureus*. The first 100 participants served as a validation cohort for the two culture method. An additional eight cultures of the anterior nares were obtained by self-sampling in these subjects. These cultures were taken approximately one week apart, spent approximately 3-6 days in transport to the University of Maryland, Baltimore, and were qualitatively tested for the presence of *S. aureus*.

We performed semi-quantitative cultures following the procedures of Nouwen et al [23]. Briefly, nasal swabs were placed in trypticase soy broth (TSB), vortexed for 15 seconds and placed in 8 ml of phenol red mannitol salt broth (PHMB). 500 ul of the original TSB suspension was placed on a phenol red mannitol salt agar plate (PHMA). A second PHMA plate was divided into 3 sections with section 1 inoculated with 10 µl of the original suspension, section 2 inoculated with 10 µl of a 1∶10 dilution of the original suspension and section 3 inoculated with 1 µl of a 1∶10 dilution of the original suspension. The plates were placed at 37C for 48 hours and then room temperature for 5 days. The PHMB was placed at 37°C for 7 days. 10 µl of the PHMB was plated onto a blood plate as well as suspect yellow colonies on the PHMA plates. *S. aureus* was confirmed using standard microbiology procedures. The number of colony-forming units were counted and reported as described previously [23]. Qualitative cultures were performed by plating the swabs directly to CHROMagar Staph aureus (Becton Dickinson, Sparks, MD). Mauve colonies were plated to a blood agar plate and S. aureus was confirmed using standard microbiology procedures. Methicillin resistance was determined using CLSI guidelines [24].

The ability to correctly determine persistent *S. aureus* colonization status with two cultures was validated via comparison against results from the self-sampled cultures, in which the presence of *S. aureus* on ≥80% of cultures was required for persistent colonization. Once it was demonstrated that the two culture method was sufficient to determine persistent *S. aureus* colonization status, the eight self-sampled, qualitative cultures were eliminated from study procedures. In the final analysis, persistent *S. aureus* colonization was defined as an average of ≥1000 CFU on the two cultures collected by the research nurse. [9].

### Statistical Methods

The sensitivity and specificity of the first two anterior nares cultures to correctly categorize persistent *S. aureus* colonization status were calculated. The association between persistent *S. aureus* colonization and potential predictors was measured using the chi square test or Fisher’s exact test for categorical variables and the Student t test for normally distributed continuous variables. The strength of the association between the semi-quantitative culture results and the qualitative culture results was measured using the Spearman rank correlation coefficient for nonparametric data.

We estimated the degree of familial aggregation by comparing the prevalence of persistent colonization in siblings of index cases who were persistent colonizers to the prevalence of persistent colonization in siblings of index cases who were not persistent colonizers. We summarized this comparison as a prevalence rate ratio. In addition, we computed the sibling relative risk as the prevalence of persistent colonization in siblings of index cases who were persistent colonizers divided by the prevalence of persistent colonization in the total population.

Finally, we used a pedigree-based maximum likelihood procedure to estimate the heritability of persistent colonization by defining heritability as the proportion of the total trait variance attributable to the additive effects of genes. Using variance component methods implemented in the SOLAR software program [25], [26], we modeled the observed phenotypic covariances between two individuals within the pedigree as having an expected value given by the product of their coefficient of relationship (which is equal to two times their kinship coefficient), the heritability, and the phenotypic variance of the trait (conditional upon covariate effects). Based on this simple model, the likelihood of the pedigree data was calculated under the assumption of multivariate normality. Parameter estimation was performed by finding those values of the parameters (including the heritability) that yielded the maximum likelihood. For dichotomous traits (e.g., persistent colonization), SOLAR assumes an underlying liability that is continuously distributed based on the threshold model.All p-values were 2-tailed and confidence intervals set at 95%. Data were analyzed using Stata (version 10.1; Stata Corporation, College Station, Texas). The initial set of 166 index cases was designed to produce a population estimate of the prevalence of persistent *S. aureus* colonization in the population as well as to generate a sample of siblings for calculation of the prevalence rate ratio. We estimated that our final sample of 232 siblings provided 80% power to detect a prevalence rate ratio of 2.25, assuming an overall prevalence rate of 20% for persistent colonization among all siblings.

## Results

A total of 400 participants were enrolled between March 2008 and October 2009. Two of these participants were subsequently withdrawn after it was discovered that they were initially ineligible (one because same-sex sibling was deceased, one because of possible skin rash present at enrollment). Of the remaining 398 participants, 100 contributed at least nine qualitative cultures for the validation study. All 398 participants contributed enough swabs to determine persistence status.

Demographic and health history characteristics are presented in Table 1. The participants had an average age of 46 years, were more often women (73% female), routinely handled animals on a daily basis (62%) and lived with multiple family members (average of 2.0 adults and 3.3 children). Participants rarely reported history of skin boils or lesions (2%), or surgery (4%), hospitalization (5%) or antibiotic use in the past year (14%).

**Table 1:** Description of study population and characteristics associated with *Staphylococcus aureus* colonization among healthy, adult Old Order Amish in Lancaster County, PA, 2008–2009*.

			*S. aureus* colonization status		
Characteristic	Category	All participants	Non-persistent	Persistent	p- value†
Sample size		398	327	71	
Age (years)		46±15	46±15	46±14	0.90
Female sex		289 (73)	236 (72)	53 (75)	0.67
Adults in household		2.0±1.4	2.0±1.4	2.1±1.4	0.57
Children in household		3.3±2.8	3.3±2.8	3.1±2.7	0.61
Routinely handle animals	Yes	247 (62)	205 (63)	42 (59)	0.66
	No	150 (38)	121 (37)	29 (41)	
	Don’t know	1 (0)	1 (0)	0 (0)	
History of skin boils or lesions		6 (2)	6 (2)	0 (0)	0.60
Surgery in last year		14 (4)	13 (4)	1 (1)	0.48
Hospitalized in last year		20 (5)	19 (6)	1 (1)	0.23
Taken antibiotics in last year		55 (14)	47 (14)	8 (11)	0.49

*Values are mean ± SD or n (%).

†P values are from chi-square test, Fisher’s exact test or t-test, as appropriate.

**Figure 1:**
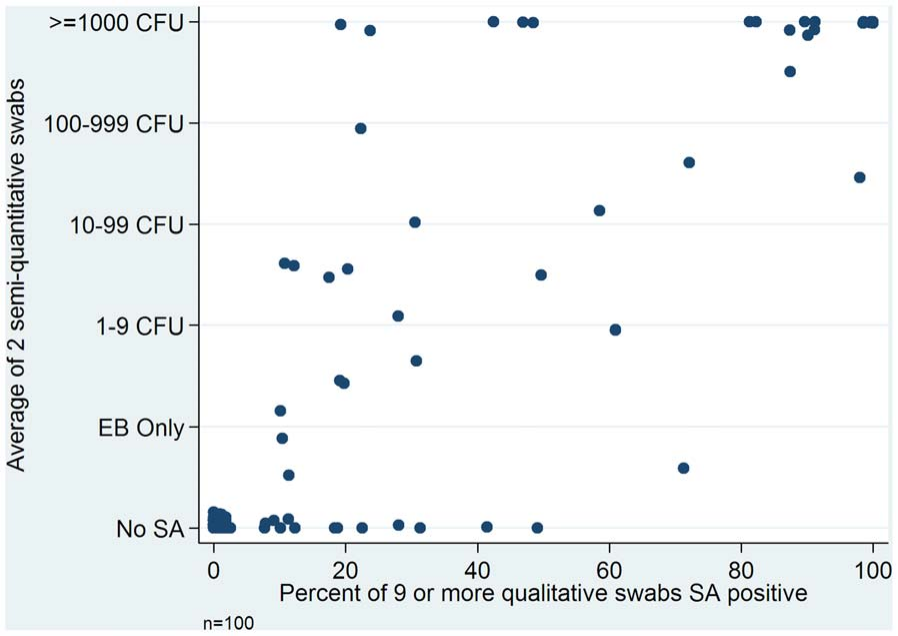
Validation of two culture method to determine nasal *S. aureus* colonization status. Correlation between the two semi-quantitative cultures for *S. aureus* and the nine or more qualitative cultures for *S. aureus* results in study population, Spearman’s rho = 0.85, p value <0.01, EB is enrichment broth, CFU is colony forming units, SA is *S. aureus*.

We validated use of two semi-quantitative cultures to define persistent *S. aureus* colonization by comparing these test results against those obtained from the use of nine or more qualitative cultures. Persistent *S. aureus* colonization was defined as an average of ≥1000 CFU on the two cultures collected by the research nurse. [9]. Of the 16 participants having *S. aureus* present on ≥80% of the cultures and therefore defined as persistent *S. aureus* carriers, 15 of them had an average of ≥1000 CFU on the first two cultures (sensitivity 94%, 95% CI 89%–98%). Of the 84 participants who did not have *S. aureus* present on ≥80% of the cultures, and were therefore not persistent *S. aureus* carriers, 79 of them had an average of <1000 CFU on the first two cultures (specificity 94%, 95% CI 89%–99%). The 2 culture rule identified 20 participants as persistent *S. aureus* carriers, 15 of whom were correctly characterized (positive predictive value 75%, 95% CI 67%–83%). Figure 1 shows the strong correlation between the average of two semi-quantitative cultures and the percent positive from nine or more qualitative culture results in our validation cohort (r = 0.85, p<0.01, Spearman’s rho).

Persistent colonization with *S. aureus* was found in 18% (71/398) of all study participants. All but two *S. aureus* isolates were methicillin susceptible. Persistent *S. aureus* colonization was not statistically significantly associated with any measured host characteristic, including age, sex or household characteristics.

Among the 166 index cases, 29 were persistent colonizers of *S. aureus* (17.5%) and 137 were not persistent colonizers. The 29 persistently colonized index cases had 36 siblings, 8 of whom (22.2%) were persistently colonized themselves. The 137 non-persistently colonized index cases had 196 siblings, 34 of whom (17.3%) were persistently colonized. This yielded a prevalence rate ratio of 1.28 (95% CI: 0.65–2.54, p = 0.64). The sibling relative risk, defined as the prevalence of persistent colonization among index cases of persistent colonizers divided by the population prevalence of persistent colonization, was 1.25 (95% CI: 0.65–2.38, p = 0.51). The heritability of persistent colonization, estimated by variance component methods, was 0.19±0.21 (p = 0.31).

## Discussion

In this familial aggregation study, we found that the trait of persistent *S. aureus* colonization does not strongly aggregate in Amish family members within different households and that heritability was low. This suggests that environmental factors (e. g. exposure to a highly colonized person or animal) or acquired host factors (e. g. nasal inflammation) are more important than host genetic factors in determining persistent *S. aureus* colonization in this community.

Colonization status is clearly influenced by multiple factors. Host factors such as age, sex, ethnicity, socioeconomic status, antibiotic use, and underlying diseases such as upper respiratory inflammation affect colonization [9], [27]. Children have higher rates of *S. aureus* colonization than adults perhaps due to a developing immune system [27]. Men have a higher risk of *S. aureus* colonization than women [18], [28]. There are different carrier rates in different ethnic groups [27], [28]. Environmental factors such as exposure to a heavily colonized individual in the household or hospital affect colonization. Household transmission studies, which have focused mainly on MRSA, have shown that transmission from MRSA colonized patients or healthcare workers occurs in 15%–29% of household contacts [29], [30]. Familial aggregation was detected in a very large (n = 11,501) community-based prevalence study in the 1960’s. There was a two-fold increase in colonization if a family member was colonized [15]; however, colonization was defined using a single culture and family members lived in the same household. Despite this, the family pairs carried similar strains less than half the time, suggesting a genetic predisposition as opposed to common household exposure. Two twin studies have failed to find a genetic component to *S. aureus* carriage; these may have been underpowered (32 identical twin pairs and 35 fraternal twin pairs in the largest study) and were done in pediatric populations who have different colonization patterns than adults [16], [17]. In our study, we controlled for household and sex by requiring sibling pairs to live in different households and to be matched on sex. We also defined *S. aureus* colonization using two cultures to distinguish between persistent and transient colonization. Other factors associated with *S. aureus* nasal carriage (age <18, ethnicity, socioeconomic status, antibiotic use, underlying diseases) were restricted via eligibility criteria. Thus we were able to control for many, though not all, of the factors known to be associated with *S. aureus* colonization. In this setting, we did not detect strong evidence for familial aggregation or heritability.

Our study population was derived from the Old Order Amish of Lancaster, Pennsylvania. They live in a rural, community-oriented environment, and represent a genetically closed homogeneous Caucasian population of Central European ancestry. Despite this, we believe our results can be generalized to a community-based Caucasian population. Most of the common genetic variants in the general Caucasian population are also represented in the Amish [31], [32]. Therefore, it is unlikely that the genetic determinants of *S. aureus* colonization in this population differ from *S. aureus* colonization in the general Caucasian population. The Amish lifestyle is different from the general population particularly in terms of routinely handling animals; however, the handling of any animals or of a specific type of animal (e.g. horses, pigs) was not associated with persistent *S. aureus* colonization (data not shown). Finally the relative frequency of persistent *S. aureus* colonization in our study population (18%) is very similar to that found in other study populations (19%), which further supports our belief that these results can be generalized to the general Caucasian population [18].

We used two semi-quantitative cultures of the anterior nares to measure our phenotype, persistent *S. aureus* colonization. We did not measure extranasal colonization, which could be a limitation; however, our method has been used to define the phenotype of persistent *S. aureus* colonization by others [18]–[23]. Our validation of this method showed excellent sensitivity, specificity and very good predictive value. Subjects self-sampled to obtain the validation cultures and these cultures were shipped via regular mail service. Although patient-collected samples have been shown have excellent agreement with provider-collected samples [33], our validation samples may have been less accurate for detecting *S. aureus* than the first two cultures which were obtained by trained research nurses and shipped overnight. Consequently our estimates of sensitivity and predictive value may be lower than if we had used similar collection and shipment methods for all of the cultures.

While we are unable to rule out the possibility of modest familial aggregation for persistent *S. aureus* colonization since our sample was powered to detect prevalence rate ratios of 2.25, our results do suggest that host genetic factors are not a strong determinant of persistent *S. aureus* colonization of the anterior nares. This implies that other potentially more modifiable factors such as environmental exposure determine *S. aureus* colonization. Environmental exposure to *S. aureus*, particularly in health care setting when patients are at increased risk for infections, can be minimized through optimal hand hygiene and antisepsis. Our results highlight the importance of current efforts to decrease the transmission of *S. aureus* in the community and healthcare settings to prevent community- and healthcare-associated *S. aureus* infections. Further research is needed to understand the acquired host and environmental factors which promote *S. aureus* colonization and their impact on transmission to others.
